# Assessment of Patient Safety Culture Among Nurses Working at Tertiary Care Hospitals in Aljouf Region, Saudi Arabia

**DOI:** 10.7759/cureus.58429

**Published:** 2024-04-16

**Authors:** Afrah S Alshammari, Tahalil Z Aldhuwayhi, Noor O Alibrahim, Shaykhah M Almhna, Zainab A Al Shehadeh, Sadeem A Altaymani, Doaa M Abdel-Salam, Rehab A Mohamed, Shimaa H Hassan

**Affiliations:** 1 Nursing, King Abdulaziz Specialist Hospital, Sakaka, SAU; 2 College of Medicine, Al Jouf University, Sakaka, SAU; 3 Department of Public Health and Community Medicine, Faculty of Medicine, Assiut University, Assuit, EGY; 4 Department of Family Medicine, Faculty of Medicine, Suez Canal University, Ismailia, EGY

**Keywords:** saudi arabia, tertiary care hospitals, nurses, safety, patient

## Abstract

Introduction: Patient safety is a fundamental element in healthcare quality and a major challenge in achieving universal health coverage, especially in low- and middle-income countries. The first step to improve patient safety is to evaluate the safety culture in hospitals. This study aimed to investigate the patient safety culture among nurses and determine the factors affecting it.

Methods: This cross-sectional study was conducted among 423 nurses working at tertiary care hospitals in the Al-Jouf region in Saudi Arabia.

Results: The highest score for patient safety among nurses was for teamwork within units (16.41 ± 2.44). The lowest score was for nonpunitive response to errors (5.87 ± 1.92). In addition, 83% of the participants did not report any events in the past 12 months. More perception of patient safety was significantly higher among females than males in dimensions of teamwork within units, frequency of events reported, and staffing. Furthermore, teamwork within units, management support for patient safety, staffing, non-punitive response to errors, and handoffs and transitions were significantly higher among participants in direct contact with patients. The Hospital Survey on Patient Safety Culture (HSOPSC) scale is significantly higher among non-Saudi nurses, nurses with bachelor's education, nurses with less working hours per week, and those who had training on patient safety.

Conclusion: The current study showed that the majority of the participants did not report any events in the past 12 months. The highest score for patient safety culture dimensions among nurses was for teamwork within units while the lowest score was for nonpunitive response to errors.

## Introduction

The World Health Organization defines patient safety (PS) as the prevention of damage to patients and emphasizes the prevention of errors, deriving lessons from mistakes, and the establishment of the healthcare delivery system based on a security culture that includes health professionals, organizations, and hospitals [1]. The European Society for Quality in Health Care defined the dynamic patient safety culture (PSC) as an individual and organizational behavior based upon shared beliefs and attitudes that continuously aim to decrease the harm of patients, which may result from the care delivery process [2]. Harm could be prevented by the involvement of healthcare professionals, organizations, and patients in preventing errors and learning from them [3].

According to the Organization for Economic Cooperation and Development report (2018), 134 million adverse events occur in hospitals annually, with about 2.6 million deaths occurring in low- and middle-income countries [4]. An “event” is defined as any type of error, mistake, incident, accident, or deviation, regardless of whether or not it results in patient harm [5]. PS addressed knowledge, skills, human resources, and systems management as the most common preventable errors related to investigations, medications, and nosocomial infections [6].

Full understanding and targeting of PS-related attitudes and behaviors are required to support a safety culture and obtain a desirable outcome in all aspects of the organization [7]. This could be done by setting up clear policies, having skilled healthcare professionals, supportive leadership, up-to-date data, and patient-centered care in order to maintain a healthcare safety culture [8].

By assessing the existing safety culture, organizations will be allowed to obtain a clear view of PS details requiring urgent attention, determine the strengths and weaknesses of their safety culture, and enhance continuous quality management [9].

Assessing the current PSC helps to get a better picture of PS issues that need immediate action, recognize the strengths and shortcomings, and improve ongoing quality control. The present study was the first to be conducted in the Al Jouf region to provide insights into PSC among nurses. The study aimed to assess PS culture among nurses working at tertiary care hospitals in the Al Jouf region, Saudi Arabia, and to explore and determine the factors affecting PS.

## Materials and methods

This cross-sectional study was conducted at five tertiary care hospitals in the Al Jouf region, Saudi Arabia (King Abdul-Aziz Specialist Hospital; Gynecology, Obstetrics and Children Hospital; Prince Mtieb Bin Abdul-Aziz Hospital; Domat Al-Jandal General Hospital; and Tabarjal General Hospital, Al-Jouf). The study included 423 nurses working at these hospitals. The study protocol was submitted to the Local Committee of Bioethics (LCBE) for approval (approval no.: 13-08/41). An informed written consent was obtained from nurses before data collection. Confidentiality of all data was assured.

The participants were either staff or charge nurses. Staff nurses provide direct, hands-on patient care, often working at their bedsides in a hospital. They often work in many different units and execute many different tasks. Charge nurses manage a shift of staff nurses, in addition to performing the patient care duties of a staff nurse. They monitor administrative and managerial tasks, such as coordinating the staffing schedule, covering call-ins, and making sure all policies are complied with.

Voluntary-participating nurses who were registered in the Saudi Commission for Health Specialties, with a license number, and had more than one year of experience were included in the present study. Refusal of participation, absent nurses, and those who did not fulfill the inclusion criteria were excluded from the present study.

The sample size was calculated using the Epi Info version 7 StatCalc, which is available from the Centers for Disease Control (CDC) and WHO. The authors determined the following criteria after reviewing the available literature to calculate the least sample size: population size of 999,999, expected frequency of 50%, confidence level of 95%, and a margin of error of 5%. The calculated sample size is 384. After adding 10% as the non-response rate, the sample size was raised to 423. The target population was selected from tertiary care hospitals in the Al Jouf region, Saudi Arabia. The number of nurses selected in each tertiary care hospital was proportional to the number of hospital beds until reaching the desired sample size.

Data were collected by distributing self-administered questionnaires in the English language as it is the main language of the staff in the hospital. The data collection tool was composed of two parts. The first part was about sociodemographic characteristics, such as age, gender, department, nationality, educational level, experience, and whether received training about patient safety or not (Appendix A).

The second part was the Hospital Survey on Patient Safety Culture (HSOPSC) scale, developed by the Agency for Healthcare Research and Quality (AHRQ) (Appendix B). It was developed in 2004 by the United States Department of Health and Human Services and became a widely used survey. This survey allows the assessment of staff opinions concerning medical errors, adverse event reporting, and other issues relevant to PS [10].

The HSOPSC includes 42 questions that assess patient safety culture across 12 basic dimensions, two outcome dimensions, and 10 safety culture dimensions (including seven unit-level dimensions and three hospital-level dimensions). The HSOPSC includes both positive and negative worded items. The response to the 10 safety culture dimensions is done using a five-point Likert scale, ranging from strongly disagree to strongly agree. The frequency of event reports filled out and submitted in the past 12 months (by a five-option scale ranging from no events to 21 events or more). The HSOPSC showed acceptable levels of internal reliability (Cronbach's alpha 0.63-0.84) and construct validity [11].

Data were analyzed using IBM SPSS Statistics for Windows, version 23.0 (released 2015, IBM Corp., Armonk, NY). Mean ± SD was calculated for 12 safety culture dimensions. Independent sample t-test and one‐way analysis of variance were conducted to show the factors that affect patient safety culture. Multivariate linear regression was done to identify the significant predictors of patient safety culture. P-value ≤ 0.05 was statistically significant.

## Results

Table 1 demonstrates the background characteristics of the participants. This study involved 423 nurses aged 24-48 years with a mean age ± SD of 31.80 ± 4.37. Most of the participants were females (91.5%) and Saudi nationals (68.6%). Nearly three-quarters of the participants were staff nurses (76.4%). In addition, 43.7% of the nurses had work experience of six to 10 years, and 95% worked 45-49 hours per week. The majority of nurses (88.4%) were in direct contact with patients and 95.7% received training on patient safety. About 83% of the participants did not report any events in the past 12 months and 11% reported one to two events in the past 12 months.

**Table 1:** Background characteristics of Saudi nurses working at tertiary care hospitals.

Background characteristics	No. (%) (n = 423)
Age	
<30 years	135 (31.9%)
30-39 years	272 (64.3%)
40-49 years	16 (3.8%)
Mean ± SD (Range)	31.80 ± 4.37 (24 - 48)
Sex	
Male	36 (8.5%)
Female	387 (91.5%)
Nationality	
Saudi	290 (68.6%)
Non-Saudi	133 (31.4%)
Education	
Bachelor	212 (50.1%)
Diploma	211 (49.9%)
Experience	
≤5 years	140 (33.1%)
6-10 years	185 (43.7%)
11-15 years	82 (19.4%)
>15 years	16 (3.8%)
Current position	
Staff nurse	323 (76.4%)
Charge nurse	100 (23.6%)
Working hours per week.	
35-39 hours/week	3 (0.7%)
40-44 hours/week	13 (3.1%)
45-49 hours/week	402 (95%)
≥50 hours/week	5 (1.2%)
Direct contact with patients	
Yes	374 (88.4%)
No	49 (11.6%)
Received training on patient safety	
Yes	405 (95.7%)
No	18 (4.3%)
Reported events in the past 12 months	
No	351(83%)
1-2 events	47 (11%)
3-5 events	17 (4%)
6-10 events	8 (2%)

Table 2 shows the mean scores of patient safety culture dimensions among the nurses working at Al Jouf tertiary care hospitals. The highest score was for teamwork within units (16.41 ± 2.44). The next highest scores were supervisor expectations and actions promoting patient safety (12.05 ± 0.58), organizational learning (12.25 ± 1.75), overall perceptions of patient safety (12.22 ± 1.06), teamwork across units (12.02 ± 1.51), and feedback and communication about error (12.05 ± 2.05). The lowest score was for nonpunitive response to errors (5.87 ± 1.92).

**Table 2:** Mean score of patient safety culture dimensions among Saudi nurses working at tertiary care hospitals.

Patient safety culture dimensions	Mean ± SD
Teamwork within units	16.41± 2.44
Supervisor expectations and actions promoting patient safety	12.05 ± 0.58
Organizational learning-continuous improvement	12.25 ± 1.75
Management support for patient safety	10.22 ± 0.97
Overall perceptions of patient safety	12.22 ± 1.06
Feedback and communication about error	12.05 ± 2.05
Communication openness	9.92 ± 1.19
Teamwork across units	12.02 ± 1.51
Frequency of events reported	11.74±2.09
Staffing	10.09 ±1.42
Handoffs and transitions	8.23 ± 2.92
Nonpunitive response to errors	5.87 ± 1.92

Table 3 reveals the correlates of patient safety culture dimensions among participants. More perception of patient safety was significantly higher among females than males in dimensions such as teamwork within units, frequency of events reported, and staffing. Furthermore, teamwork within units, management support for patient safety, staffing, non-punitive response to errors, and handoffs and transitions were significantly higher among participants in direct contact with patients. The charge nurse had a significantly higher perception of patient safety than the staff nurse in dimensions, such as staffing, and non-punitive response to errors.

**Table 3:** Correlates of the mean scores of patient safety culture dimensions among Saudi nurses working at tertiary care hospitals.

Patient safety culture dimensions	Gender		P-value	Direct contact with patients		P-value	Current position		P-value
	Male	Female		Yes	No		Staff nurse	Charge nurse	
Teamwork within units	16.25 ± 2.42	18.11 ± 1.95	0.000	16.51 ± 2.41	15.69 ± 2.57	0.028	16.46 ± 2.48	16.25 ± 2.33	0.450
Supervisor expectations and actions promoting patient safety	12.03 ± 0.61	12.05 ± 0.58	0.834	12.03 ± 0.55	12.14 ± 0.79	0.357	12.03 ± 0.58	12.10 ± 0.59	0.301
Organizational learning, continuous improvement	12.19 ± 2.71	12.26 ± 1.63	0.890	12.29 ± 1.69	11.92 ± 2.12	0.154	12.32 ± 1.69	12.05 ± 1.91	0.184
Management support for patient safety	10.47 ± 1.56	10.19 ± 0.89	0.302	10.28 ± 0.96	9.78 ± 0.92	0.001	10.05 ± 0.96	10.27 ± 0.97	0.075
Overall perceptions of patient safety	12.58 ± 1.46	12.18 ± 1.01	0.114	12.21 ± 1.05	12.29 ± 1.15	0.620	12.22 ± 1.05	12.20 ± 1.10	0.870
Teamwork across units	11.69 ± 2.31	12.05 ± 1.41	0.372	12.02 ± 1.57	11.98 ± 0.97	0.846	12.03 ± 1.53	11.97 ± 1.42	0.711
Feedback and communication about error	11.47 ± 3.40	12.10 ± 1.88	0.276	12.12 ± 1.98	11.53 ± 2.48	0.113	12.14 ± 1.94	11.77 ± 2.36	0.154
Communication openness	9.78 ± 1.85	9.93 ± 1.11	0.630	9.93 ± 1.19	9.84 ± 1.19	0.615	9.90 ± 1.27	9.97 ± 0.88	0.612
Frequency of events reported	9.81 ± 3.18	11.92 ± 1.87	0.000	11.82 ± 2.07	11.14 ± 2.24	0.069	11.35 ± 2.25	11.86 ± 2.04	0.063
Staffing	9.97 ± 1.23	11.39 ± 2.37	0.001	10.84 ± 1.72	9.99 ± 1.35	0.002	9.94 ± 1.34	10.55 ± 1.59	0.001
Handoffs and transitions	8.69 ± 3.58	8.19 ± 2.85	0.415	9.14 ± 3.29	8.11 ± 2.85	0.041	8.09 ± 2.83	8.67 ± 3.17	0.107
Non-punitive response to errors	5.58 ± 2.69	5.89 ± 1.84	0.503	6.67 ± 2.18	5.76 ± 1.86	0.007	5.74 ± 1.77	6.28 ± 2.32	0.034

Table 4 reveals the correlation between different patient safety culture dimension scores. Nearly each safety culture dimension had a positive correlation with the other dimensions. Table 5 illustrates that the HSOPSC is significantly higher among non-Saudi nurses (0.028), nurses with bachelor's education (0.001), nurses with less working hours per week (0.000), and those who had training on patient safety (0.003).

**Table 4:** Correlation between patient safety culture dimensions among Saudi nurses working at tertiary care hospitals. **Correlation is significant at the 0.01 level (2-tailed). *Correlation is significant at the 0.05 level (2-tailed).

	Teamwork within units	Supervisor expectations and actions promoting patient safety	Organizational learning, continuous improvement	Management support for patient safety	Overall perceptions of patient safety	Teamwork across units	Feedback and communication about error	Communication openness	Frequency of events reported	Staffing	Handoffs and transitions	Non-punitive response to errors
Teamwork within units	1	-	-	-	-	-	-	-	-	-	-	-
Supervisor expectations and actions promoting patient safety	.148**	1	-	-	-	-	-	-	-	-	-	-
Organizational learning, continuous improvement	.586**	.072	1	-	-	-	-	-	-	-	-	-
Management support for patient safety	.422**	.321**	.459**	1	-	-	-	-	-	-	-	-
Overall perceptions of patient safety	-.039	.383**	.090	.412**	1	-	-	-	-	-	-	-
Teamwork across units	.345**	.366**	.219**	.371**	.296**	1	-	-	-	-	-	-
Feedback and communication about error	.379**	-.089	.580**	.337**	.003	.222**	1	-	-	-	-	-
Communication openness	.163**	.095	.255**	.211**	.061	.088	.461**	1	-	-	-	-
Frequency of events reported	.428**	.012	.611**	.186**	-.185**	.209**	.628**	.517**	1	-	-	-
Staffing	-.438**	.196**	-.540**	-.242**	.186**	-.120*	-.540**	-.324**	-.765**	1	-	-
Handoffs and transitions	-.493**	.208**	-.494**	-.027	.327**	.038	-.399**	-.258**	-.668**	.650**	1	-
Non-punitive response to errors	-.595**	.109*	-.502**	-.168**	.227**	.037	-.356**	-.086	-.478**	.501**	.722**	1

**Table 5:** Linear regression model demonstrating the predictors of the Hospital Survey on Patient Safety Culture (HSOPSC) scale among Saudi nurses working at tertiary care hospitals. Reference groups: sex (males), nationality (Saudi), education (diploma), current position (staff nurse), direct contact with patients (no), received training on PS (no)

Variables	Beta	t	p-value	95% confidence interval	
				Lower limit	Upper limit
Age	0.285	1.251	0.959	-0.334	0.752
Sex (females)	-0.342	-1.761	0.447	-0.962	0.041
Nationality (non-Saudi)	0.147	2.199	0.028	0.042	0.287
Education (bachelor)	4.250	15.356	0.001	10.482	12.674
Experience	0.370	0.641	0.522	-0.255	0.502
Current position (charge nurse)	0.008	1.476	0.149	-0.054	0.042
Working hours per week	-0.234	-2.640	0.000	-0.627	0.283
Direct contact with patients (Yes)	0.362	2.435	0.352	-0.346	0.652
Received training on PS (Yes)	0.052	3.855	0.003	0.02	0.009

## Discussion

Staff organization commitment to patient safety, seeking safety in the face of barriers, and willingness to disclose near-misses and bad incidents all contribute to the organization's safety culture. This also shows individuals' and organizations' ability to deal with risks and dangers, avoid errors, and achieve their objectives [12].

This study aimed to assess patient safety culture among nurses working in tertiary care in the Al Jouf region, Saudia Arabia. The study used the HSOPSC scale, which is a valid and reliable method for assessing the patient safety culture in hospitals.

The study's findings revealed that nurses working in the facility valued PSC more regarding teamwork within units that had the highest score (16.41 ± 2.44). This means that they like active performance and cooperation with their colleagues in the same unit. In addition, this indicates that teamwork among units is a hospital's strength strategy for creating a positive and safe environment for patients. Moreover, this component is important for providing safe and effective care because treatment is typically provided by a multidisciplinary team in several locations around the hospitals.

Similarly, the score of teamwork within units was the highest reported in Saudi Arabia [13]. This was also consistent with other studies at a tertiary government hospital in the Philippines [14], Egypt [15-17], India [18, 19], Nepal [20], and primary healthcare units in Tunisia [21].

By contrast, another study recorded "teamwork within units" with the lowest positive response score of 21.4% [22]. This can be explained by the existence of different policies and procedures for communication within Saudi organizations and the different leadership styles of nurse managers. In addition, teaching hospitals have teams with a variety of experiences and communication problems with junior doctors, which can lead to the “lack of shared mental models,” resulting in poor teamwork. Regarding this, supportive leadership, teamwork, and professional communication training should be emphasized as an effective strategy for ensuring a patient safety culture.

On the other hand, the lowest documented score in this study was for nonpunitive responses to errors (5.87 ± 1.92). This finding indicated that the nurses were feeling scared of reporting errors. They also may be vulnerable to blame and penalties in the hospital. The hesitation of the nurses to report adverse events may be due to the pervasiveness of a punitive response to error and blame culture, documentation of errors, and fear of the consequences. All lead to reduced frequency of error reporting among nurses. For a positive patient safety culture, a blame-free, fear-free, and error-reporting environment should be imposed. This finding was in agreement with other studies in India, Saudi Arabia, Pakistan, and Afghanistan [19,22-25].

Next to teamwork within units, the participants reported high scores for organizational learning-continuous Improvement (12.25 ± 1.75). As governmental hospitals in Saudi Arabia have a unit for continuing education, which values and supports the educational support and learning of staff, organizational learning is a strength in Saudi Arabia’s patient safety culture [26]. Furthermore, in Saudi Arabia, nurses should complete their continuing medical education hours for the renewal of their licenses [27].

Patient safety can be achieved when all have a common background knowledge of roles and responsibilities to perform essential functions, communicate, and transfer the ﬂow of required information. Moreover, patient safety can be ensured by individual professional learning, interprofessional team learning, and system-based organizational learning. This finding was in line with those of other studies [15,22,25].

The composite mean scores of the participants in this survey were higher for teamwork within units (16.41 ± 2.44) as compared with teamwork across units (12.02 ± 1.51). Teamwork across units means the extent to which hospital units cooperate and coordinate with one another to provide the best care for patients, while teamwork within units means the extent to which staff supports each other, treat each other with respect, and work together as a team [10]. This finding requires further exploration in the future as interdepartmental friction is often a barrier to the obtainment of optimum patient safety. As the patient is often treated by several specialists in multidisciplinary healthcare facilities, cooperation and open communication should be practiced across all units. This result was the same in a tertiary care hospital in Pakistan [24].

In the present study, the domains hands oﬀ and transitions and communication openness, which require cooperation from other departments, showed low mean scores of 8.23 ± 2.92 and 9.92 ± 1.19, respectively. This indicates that there are problems in the exchange of information and ineffective communication inside or across hospital units, which in turn could adversely affect patient safety. In this study, about one-third of the nurses were non-Saudi nationals (31.4%). This multicultural nature of nurses with different cultural beliefs, languages, religions, and nationalities may risk effective communication.

This was compatible with other studies in Saudi Arabia that reported communication and language barriers between healthcare providers, mainly between Saudi nurses and non-Saudi nurses, to affect health services coordination and planning, thus introducing another risk to patient safety due to miscommunication [19,23,28,29].

Regarding the number of reported events, 83% of the participants did not report any events in the past 12 months. This means that mistakes that harmed or had the potential to harm patients were less frequently reported due to fear of the consequences of event reporting. Previous studies report a lower rate of non-reporting of events in Saudi Arabia (43%, 41%, 44%) [22,23], the Philippines (71.48%) [14], Tunisia (71%) [21], Afghanistan (49.8%) [25], and Egypt (12.1%, 43.6%) [15].

This was in line with the mean score of the frequency of events reported dimension (11.74 ± 2.09). This practice of little or no reporting of events may be due to the hesitation of healthcare staff to report adverse events owing to their perception that their mistakes would be held against them and that such mistakes would be recorded in their file. In addition, errors are always seen as a lack of skill not as a learning opportunity. Other barriers to reporting errors include loss of patient confidence, insufficient time to report, and absence of feedback.

Reporting of adverse events is an integral part of the continuous cycle of quality improvement to enhance patient safety that includes error identification, reporting, analysis, and corrective actions. It is essential to enable a supportive environment to identify and report adverse events without threat of punishment or blame. Such an environment will encourage nurses to report adverse events for the sake of patient safety and risk reduction. The frequency of events reported was also the dimension with the lowest score in other studies [16,21].

Another area that showed problems was the staffing dimension with a score of 10.09 ± 1.42. The shortfall of staff is a big challenge for the hospital, and staff is an essential element of patient safety. This study found that 96.2% of the participants worked more than 45 hours per week. This workload causes chronic fatigue, increasing the possibility of errors and side effects on patient safety. This was consistent with previous studies that reported low scores in the staffing dimension [22,24,25].

Decision makers cannot base their decisions only on a perceived adequacy of staffing. They should understand what shapes staffing adequacy to tailor strategies and take actions appropriate to the context of the organization. The perceived adequacy of staffing can be affected by several factors not only the actual shortage of staff, such as poor organization and coordination of work and underutilization of technologies, which can reduce the burden of patient care issues [30].

In the current study, the perception of patient safety was significantly higher in females than males in dimensions, such as teamwork within units, frequency of events reported, and staffing. This was inconsistent with previous studies conducted in Egypt and in Jeddah, Saudi Arabia [15,23]. This difference may be attributed to the differences in the female-to-male ratio, which was higher in the current study (10:1). In general, females demonstrated better patient safety perception as compared to males. This may be attributed to the caring nature of females, making them more sensitive to patient safety than males. Gender significantly directly affects both managerial and professional aspects of patient safety.

The results of the present study revealed that nurses’ age, gender, working experience, current position, and direct contact with patients showed no significant difference in the overall perceived patient safety culture. On the other hand, the HSOPSC scale was significantly higher among non-Saudi nurses (0.028), nurses with bachelor's education (0.001), nurses with less working hours per week (0.000), and those who had training on patient safety (0.003). These findings were compatible with another study conducted in Saudi Arabia [23].

As nationality, educational level, less working hours per week, and receiving training on patient safety were the significant predictors of the perceived patient safety culture of nurses, patient safety culture measurements should consider the interaction between organizational and individual factors, which could provide a better understanding of group dynamics and individual attitudes toward patient safety culture.

Future studies are recommended with a longitudinal design to identify the effect of time in reporting patient safety culture. Further research is also required to include the perspectives of other staﬀ in the hospital. Policy-makers and nurse leaders should develop appropriate standards for patient safety systems. This can be achieved by building leadership capacity that supports open communication, blame-free environment, teamwork, and continuous organizational learning. Building a safety culture requires eliminating three destructive elements in organizations: fear, blame, and silence regarding errors.

Regarding study limitations, the current study was carried out among nurses in tertiary care hospitals only, so that results cannot be generalized to other institutions and other categories of hospital staff as physicians and employees. The study was cross-sectional, so it only represented a snapshot of the perceptions of patient safety at a single point in time. Collected data were self-reported and may be subjected to recall bias, especially when reporting the number of adverse events, resulting in possible under- or overestimation of findings. A self-administered questionnaire may also give desirable answers that do not reflect reality for fear of revenge or proceedings. In addition, using the Likert-type scale, nurses may simply mark the two extremes of the scale throughout the questionnaire, so the true spectrum of perspectives may not have been captured.

However, the present study had some strengths as it used a well-validated questionnaire that has been widely used in many studies, including that conducted in Saudia Arabia with a large sample size, which yielded valid results.

## Conclusions

In this study, the participants valued the aspects of teamwork within units, organizational learning-continuous improvement, and overall perceptions of patient safety as areas of strength and important elements of patient safety culture. On the other hand, we highlighted different areas of concern that need improvement, such as nonpunitive response to errors, handoffs and transitions, communication openness, staffing, and frequency of events reported. More attention should be paid to these elements because changing values, behaviors, and attitudes needs time, motivation, and training to improve risk management skills among nurses.

## Appendices

Appendix A

This questionnaire aims to assess patient safety culture among nurses working at tertiary care hospitals in the Al Jouf region, Saudi Arabia. The questionnaire is distributed anonymously (without names). Privacy and confidentiality of data will be assured. Thanks in advance for your participation.

**Table 6:** First part of the survey (sociodemographic characteristics)

1	Age	
2	Gender	1 - Male 2 - Female
3	Department	
4	Direct contact with patients	1 - Yes 2 - No
5	Nationality	1 - Saudi 2 - Non-Saudi
6	Educational level	1 - Diploma 2 - Bachelor 3 - Master’s degree
7	Experience in years	
8	Current nursing position	1 - Staff nurse 2 - Charge nurse 3 - others
9	Number of working hours per week	
10	Number of reported events in the last 12 months	
11	Received training related to patient safety	1 - Yes 2 - No

Appendix B 

**Table 7:** Second part of the survey (Hospital Survey on Patient Safety Culture (HSOPSC) scale)

Strongly agree	agree	Neutral	disagree	Strongly disagree		
Teamwork within units						
					People support one another in this unit.	12
					When a lot of work needs to be done quickly, we work together as a team to get the work done	13
					In this unit, people treat each other with respect	14
					When one area in this unit gets really busy, others help out.	15
Supervisor/manager expectations and actions promoting patient safety						
					My supervisor/manager says a good word when he/she sees a job done according to established patient safety procedures.	16
					My supervisor/manager seriously considers staff suggestions for improving patient safety.	17
					Whenever pressure builds up, my supervisor/manager wants us to work faster, even if it means taking shortcuts.	18
					My supervisor/manager overlooks patient safety problems that happen over and over.	19
Organizational learning-continuous improvement						
					We are actively doing things to improve patient safety.	20
					Mistakes have led to positive changes here.	21
					After we make changes to improve patient safety, we evaluate their effectiveness.	22
Management support for patient safety						
					Unit management provides a work climate that promotes patient safety.	23
					The actions of unit management show that patient safety is a top priority.	24
					Unit management seems interested in patient safety only after an adverse event happens	25
Overall perceptions of patient safety						
					Patient safety is never sacrificed to get more work done.	26
					Our procedures and systems are good at preventing errors from happening.	27
					It is just by chance that more serious mistakes don't happen around here.	28
					We have patient safety problems in this unit	29
Teamwork across units						
					There is good cooperation among units that need to work together.	30
					Units work well together to provide the best care for patients	31
					Units do not coordinate well with each other	32
					It is often unpleasant to work with staff from other units	33
Feedback and communication about error						
					We are given feedback about changes put into place based on event reports.	34
					We are informed about errors that happen in this unit.	35
					In this unit, we discuss ways to prevent errors from happening again.	36
Communication openness						
					Staff will freely speak up if they see something that may negatively affect patient care.	37
					Staff feels free to question the decisions or actions of those with more authority	38
					Staff is afraid to ask questions when something do not seem right.	39
Frequency of events reported						
					When a mistake is made, but is caught and corrected before affecting the patient, how often is this reported?	40
					When a mistake is made, but has no potential to harm the patient, how often is this reported?	41
					When a mistake is made that could harm the patient, but does not, how often is this reported?	42
Staffing						
					We have enough staff to handle the workload	43
					Staff in this unit works longer hours than is best for patient care	44
					We use more agency/temporary staff than is best for patient care	45
					We work in "crisis mode" trying to do too much, too quickly	46
Handoffs and transitions						
					Things "fall between the cracks" when transferring patients from one unit to another.	47
					Important patient care information is often lost during shift changes	48
					Problems often occur in the exchange of information across primary healthcare service	49
					Shift changes are problematic for patients in primary healthcare service	50
Non-punitive response to errors						
					Staff feels like their mistakes are held against them	51
					When an event is reported, it feels like the person is being written up,not the problem	52
					Staff worry that mistakes they make are kept in their personnel file	53
